# CD69 Does Not Affect the Extent of T Cell Priming

**DOI:** 10.1371/journal.pone.0048593

**Published:** 2012-10-30

**Authors:** Elisenda Alari-Pahissa, Laura Notario, Elena Lorente, Javier Vega-Ramos, Ana Justel, Daniel López, José A. Villadangos, Pilar Lauzurica

**Affiliations:** 1 Instituto de Salud Carlos III, Majadahonda, Madrid, Spain; 2 Departament de Fisiologia, Universitat de Barcelona, Barcelona, Spain; 3 Department of Microbiology and Immunology, and Department of Biochemistry and Molecular Biology (Bio21 Institute), University of Melbourne, Melbourne, Australia; 4 Facultad de Matemáticas, Universidad Autónoma de Madrid, Madrid, Spain; National Institute of Allergy and Infectious Diseases, United States of America

## Abstract

CD69 is rapidly upregulated on T cells upon activation. In this work we show that this is also the case for CD69 expression on dendritic cells (DC). Thus, the expression kinetics of CD69 on both cell types is reminiscent of the one of costimulatory molecules. Using mouse models of transgenic T cells, we aimed at evaluating the effect of monoclonal antibody (MAb)-based targeting and gene deficiency of CD69 expressed by either DC or T cells on the extent of antigen (Ag)-specific T cell priming, which could be the result of a putative role in costimulation as well as on DC maturation and Ag-processing and presentation. CD69 targeting or deficiency of DC did not affect their expression of costimulatory molecules nor their capacity to induce Ag-specific T cell proliferation in *in vitro* assays. Also, CD69 targeting or deficiency of transgenic T cells did not affect the minimal proliferative dose for different peptide agonists *in vitro*. In *in vivo* models of transgenic T cell transfer and local Ag injection, CD69 deficiency of transferred T cells did not affect the extent of the proliferative response in Ag-draining lymph nodes (LN). In agreement with these results, CD69 MAb targeting or gene deficiency of Vaccinia-virus (VACV) infected mice did not affect the endogenous formation of virus-specific CD8^+^ T cell populations at the peak of the primary immune response. Altogether our results argue against a possible role in costimulation or an effect on Ag processing and presentation for CD69.

## Introduction

CD69 is a type II C-type lectin of unknown ligand specificity encoded in the NK-complex. It is known as a very early activation marker, since it is promptly upregulated on all leukocytes upon activation [1]–[2]. Importantly, it is upregulated on T cells by IFNα/β [3], and upon Ag encounter [4]–[5], during the first kinetics phase of brief contacts between T cells and antigen presenting cells, either in the presence or absence of adjuvant [6]. CD69 expression has been reported in infections [7]–[10], autoimmune diseases [11]–[16], and tumor infiltrates [17]–[18].

Some C-type lectins are upregulated on T cells upon activation and have costimulatory or coinhibitory effects, influencing the extent of TCR-mediated T cell activation [19]–[20]. Apart from that, most C-type lectin receptors are expressed by DC [21], and some of them have been shown to induce signaling or to influence Toll-like receptors (TLR)-induced signaling, modulating the maturation status of the DC [22]. This can affect their Ag processing and presentation activity as well as surface expression of co-stimulatory molecules and cytokine production, all of which can influence the capacity of the DC for priming Ag-specific T cells.

Traditionally, a costimulatory role was attributed to CD69, since anti-CD69 monoclonal antibody (MAb) treatment of pre-activated human leukocytes led to further activation. In the case of T cells, the addition of anti-CD69 MAbs enhanced anti-CD3 and PMA-induced proliferation [23]–[25] through increased interleukin (IL)-2 and IL-2 receptor expression [26] [4]. However a later study using CD69^−/−^ mice argued against such a role, since Ag-specific T cell proliferation was unaffected *in vivo* [27]. Even more in contrast, our group proposed a negative regulatory role for CD69, since tumor-bearing CD69^−/−^ mice showed increased anti-tumor immunity to NK sensible tumors [28]. That was consistent with later *in vivo* results showing that CD69^−/−^ mice had increased incidence and severity of different T cell-dependent autoimmune and inflammatory diseases such as Collagen II Induced Arthritis [29], allergic asthma, skin contact hypersensitivity [30] and autoimmune myocarditis [31]. CD69^−/−^ mice also showed increased susceptibility to *Listeria monocytogenes* (Lm) infection, associated with enhanced type I and II interferon (IFN) responses [10]. Interestingly, in the tumor, arthritis and contact hypersensitivity models, the *in vivo* treatment with the anti-CD69 2.2 MAb also led to increased anti-tumor [32], autoimmune [33] and inflammatory responses [30]. However, this antibody has agonist activity, since it induces a variety of downstream functional outcomes in purified cell types, like IFNγ secretion in NK cells [32], IL-2 secretion in plasmacytoid DC [34], CD25 upregulation in IL-2-treated T cells [34] and TGFβ secretion when crosslinked on anti-CD3-activated T cells [28]. *In vivo*, anti-CD69 2.2 MAb treatment, but not CD69 gene deficiency, induced bystander proliferation of memory-phenotype T cells [34].

**Figure 1:**
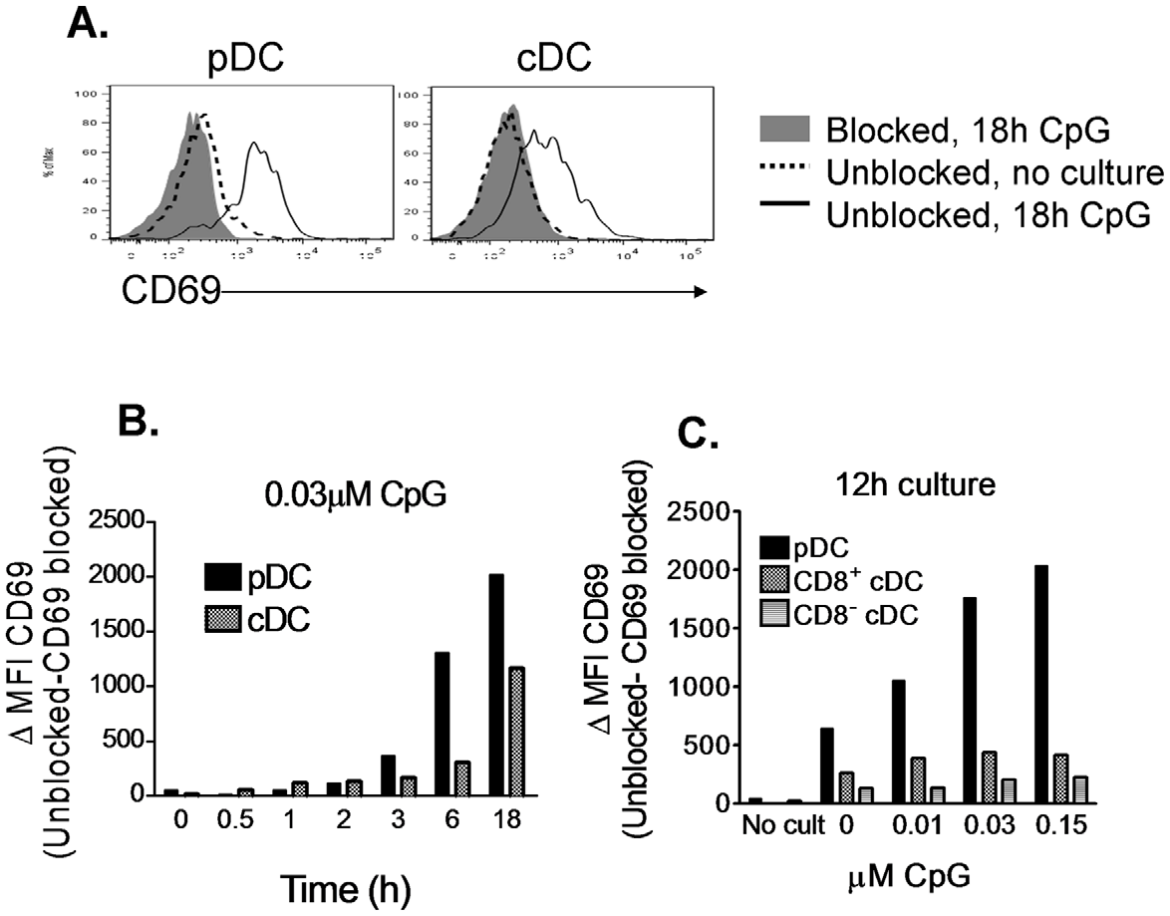
CD69 is upregulated on endogenous DC upon activation. **A.–C.** DC were purified from spleens of C57BL/6 mice and cultured with CpG in various conditions. 10 γg/ml of anti-CD69 2.2 were added to control samples in order to block CD69 staining and provide a background staining control. 10 µg/ml of Isotype control Ab were added to test samples. All samples were stained for the different DC subsets markers and for CD69, and analyzed by flow cytometry. **A.** Overlay of CD69 expression between CD69 blocked control samples cultured with 0.03 µM CpG for 18h (grey filled), and unblocked samples, uncultured (dashed line) or cultured with 0.03 µM CpG for 18h (solid line), gated on pDC (CD11c^int^, CD45RA^+^), and cDC (CD11c^hi^, CD45RA^−^). **B.** DC were cultured with 0.03 µM CpG during various time-spans and CD69 was assessed in pDC (CD11c^int^, CD45RA^+^) and cDC (CD11c^hi^, CD45RA^−^). **C.** pDC (CD11c^int^, CD45RA^+^), CD8^+^ cDC (CD11c^hi^, CD45RA^−^, CD8^+^) and CD8^−^ cDC (CD11c^hi^ , CD45RA^−^, CD8^−^) were analyzed for CD69 expression after 12h culture with different doses of CpG or without having been cultured. In B and C, CD69 levels are expressed as the difference of CD69 MFI between the unblocked and blocked samples. Results representative of two similar experiments are shown.

In this study, we revisit the question of a role for CD69 on T cell costimulation and priming, which could contribute to the observed effects of CD69 on the different T cell-dependent immune responses. To this effect we use antibody-based targeting and gene knock-out approaches in *in vitro* and *in vivo* transgenic T cell mouse models as well as viral infection models. We expand the study upon a possible effect of CD69 on the extent of T cell priming, not only from its expression on T cells, but also from its expression on the other cell type participating in T cell priming, the dendritic cells. Our results point to that CD69 does not affect the extent of T cell priming, suggesting that it does not function as a costimulatory molecule, and that it does not affect Ag presentation.

**Figure 2:**
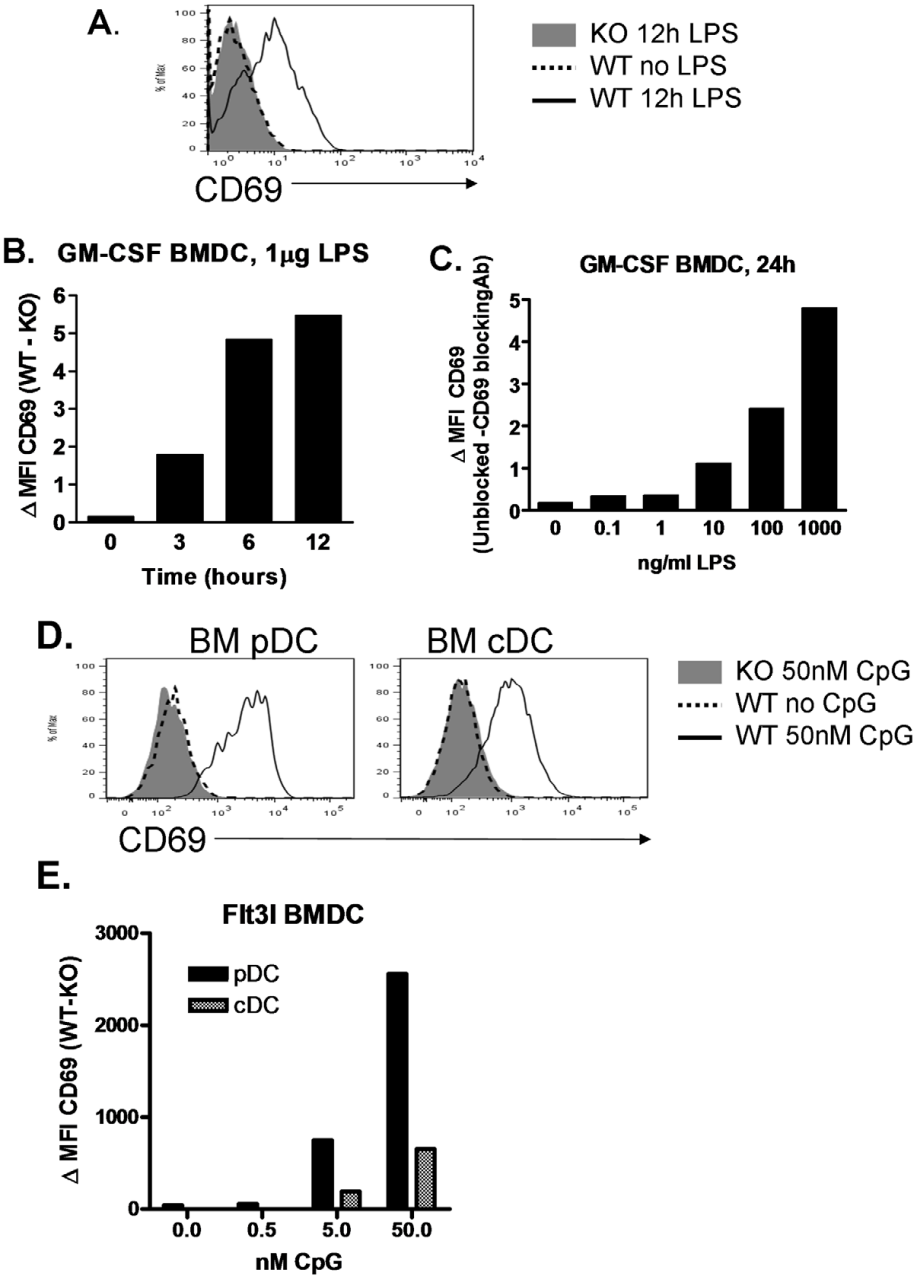
CD69 is upregulated on BMDC upon activation. CD69^+/+^ or CD69^−/−^ GM-CSF BMDC were treated with LPS at different conditions of time and dose. Subsequently they were stained for CD11c and CD69, and analyzed by flow cytometry. **A.** Overlay of CD69 histograms of CD69^+/+^ untreated cells (dashed line), and CD69^–/−^ (grey filled) and CD69^+/+^ (solid line) cells cultured for 12h with 1 µg/ml of LPS (right). **B.** Time course of CD69 upregulation in response to 1 µg/ml of LPS. CD69 expression is the difference in MFI between CD69^+/+^ and CD69^−/−^ BMDC. **C.** CD69^+/+^ BMDC were cultured for 24h with growing doses of LPS and with anti-CD69 2.2 (to provide a background staining control) or Isotype control. CD69 surface expression is expressed as the difference in MFI between unblocked and blocked samples. Results are representative of two experiments with similar results. **D.** and **E.** CD69^+/+^ or CD69^−/−^ Flt3l-derived BMDC were treated with various CpG doses for 24h and stained for the different DC subset markers and CD69. **D.** Overlay of the CD69 histograms of 50nM CpG-treated CD69^−/−^ (grey filled), and untreated (dashed line) or 50nM CpG-treated (black line) CD69^+/+^ pDC (CD11c^+^, CD45RA^+^, left) and cDC (CD11c^+^, CD45RA^−^, right). **E.** CD69^+/+^ and CD69^−/−^ BMDC were cultured with growing doses of CpG for 24h. CD69 surface levels are expressed as the difference in MFI between CD69^+/+^ and CD69^−/−^ BMDC. The results shown are of one out of two similar experiments.

## Materials and Methods

### Mice

Balb/c, DO10.11 RAG2−/− Balb/c, C57BL/6 and OT-I C57BL/6 mice, all both CD69^+/+^ and CD69^−/−^, and OT-I RAG1^−/−^ C57BL/6, OT-II C57BL/6 and H-2 class I knockout HLA-A*0201-transgenic [35] mice were bred and housed under specific pathogen free conditions in the animal facilities of the *Parc Cientific de Barcelona*, Barcelona, *Instituto de Salud Carlos III*, Madrid, and the Walter and Eliza Hall Institute, Melbourne. CD69^−/−^ mice had been backcrossed on the C57BL/6 and the Balb/c backgrounds at least nine times [27]. All procedures involving animals and their care were approved by the University of Barcelona and ISCIII Ethics Committees and were conducted according to institutional guidelines in compliance with local (Generalitat de Catalunya decree 214/1997, DOGC 2450) and international (Guide for the Care and Use of Laboratory Animals, NIH 85–23, 1985) laws and policies. Unless otherwise stated, C57BL/6 mice were used.

**Figure 3:**
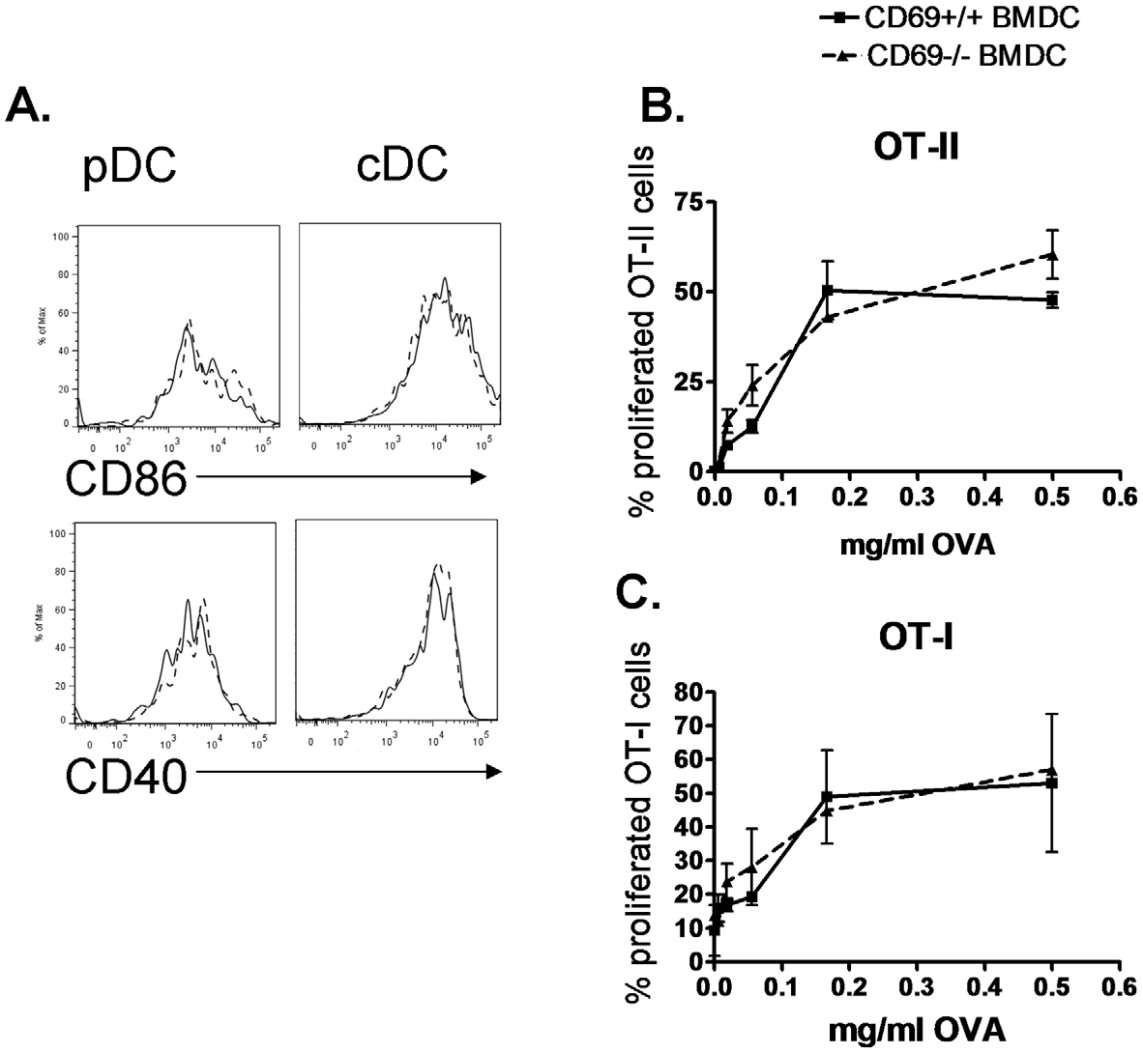
CD69 deficient DC do not have altered priming capacity. **A.** CD69^+/+^ or CD69^−/−^ Flt3l-derived BMDC were cultured with 0.5 µM CpG for 24 h. Overlays of CD69^+/+^ (solid line) and CD69^−/−^ (dashed line) Flt3l-derived BMDC showing CD86 and CD40 expression on pDC (CD11c^+^, CD45RA^+^, left) and cDC (CD11c^+^, CD45RA^−^, right). **B.** and **C.** CD69^+/+^ or CD69^−/−^ Flt3l-derived BMDC were pulsed with OVA at the indicated doses for 45 minutes, washed, and further cultured with OT-II (**B.**) or OT-I (**C.**) T cells for 3 or 2 days, respectively, in the presence of 0.5 µM CpG in duplicate. Percentage of proliferated Vα2^+^ CD4^+^ or Vα2^+^ CD8^+^ cells is depicted. Bars represent Standard Deviation (SD) of duplicate cultures. Experiments representative of two with similar results.

**Figure 4:**
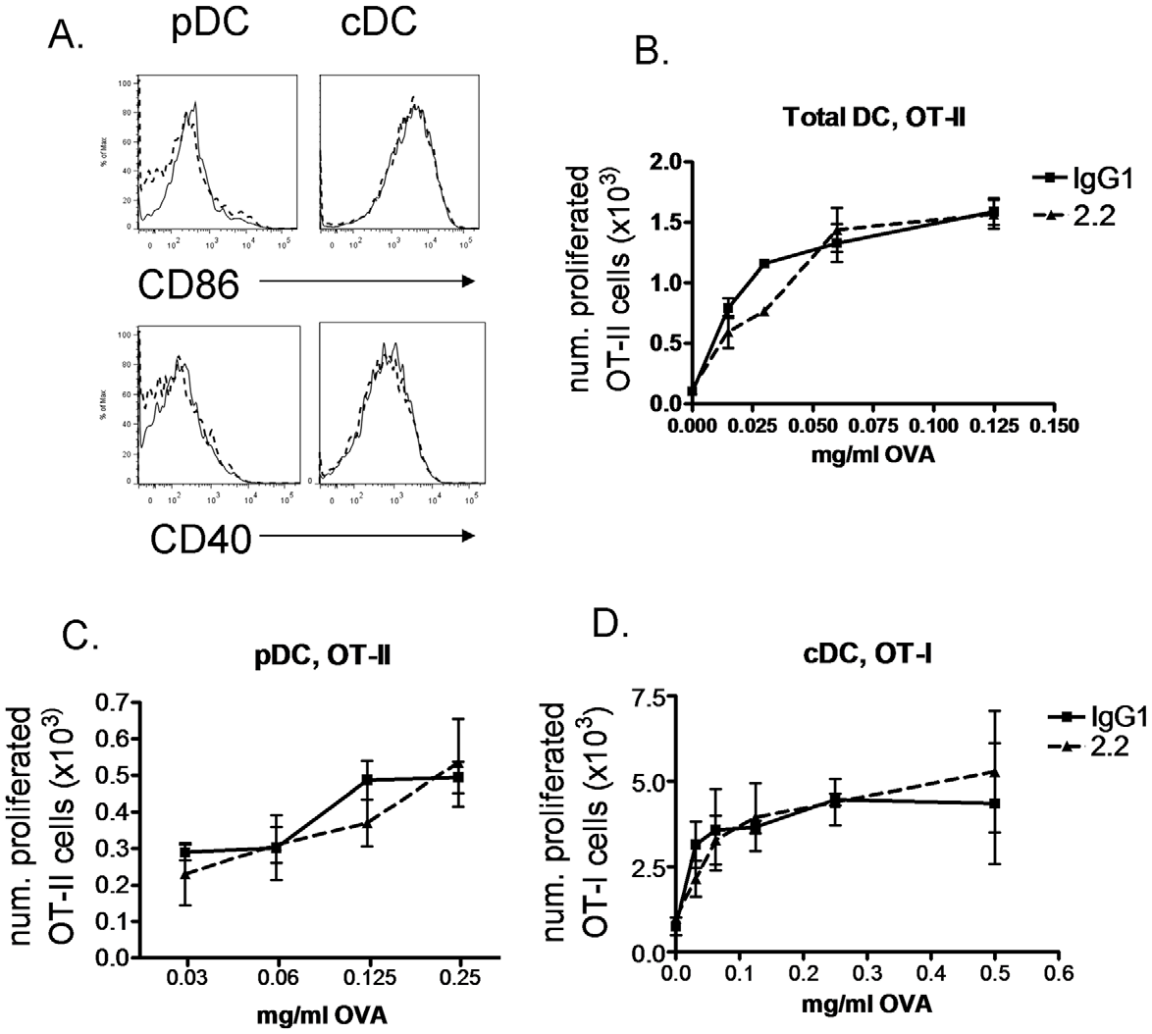
CD69 targeting on DC does not alter their costimulation and priming capacity. **A.** Flt3l BMDC were cultured with 50 nM CpG and anti-CD69 2.2 (solid line) or IgG1 control (dashed line) for 24 h. CD86 and CD40 expression levels were determined on pDC (CD11c^+^, CD45RA^+^, left) and cDC (CD11c^+^, CD45RA^−^, right). Result representative of two experiments. **B.** Purified DC were cultured with OT-II CD4^+^ T cells, in the presence of the indicated OVA doses, 0.03 µM CpG and 10 µg/ml anti-CD69 2.2 or IgG1 isotype control, in duplicate, for 3 days. **C.** Sorted pDC were cultured with the indicated OVA doses and 10 µg/ml anti-CD69 2.2 or IgG1 isotype control for 1 h. After wash, they were co-cultured with CD4^+^ OT-II T cells for 3 days. **D.** Sorted cDC were pulsed with the indicated OVA doses for 45 minutes and treated with 0.025 µM CpG and anti-CD69 2.2 or IgG1 control for 18 h. Then, they were cultured with OT-I CD8^+^ T cells for 2 days. In all cases, the number of divided cells within Vα2^+^ CD8^+^ or Vα2^+^ CD4^+^ live cells is represented. Bars represent SD of duplicate cultures.

### Antibodies and immunological reagents

The anti-CD69 2.2 mAb (IgG1 isotype) was generated in our laboratory [32] by the fusion of NS-1 myeloma cells with spleen cells from a CD69^−/−^ mouse previously immunized three times with mouse 300–19 pre-B cells. The Ab was purified from concentrated hybridoma supernatants using a protein G column (GE Healhtcare, Piscataway, NJ, USA), dialyzed extensively against PBS, further purified by high-performance liquid chromatography (HPLC) using a Superdex 200 column (GE Healhcare,) and stored at −80°C. The IgG1 isotype control antibody was produced and purified likewise. The resulting antibody preparations were tested on CD69^−/−^ bone marrow-derived DC (BMDC) cultures at 10 μg/ml, and were unable to upregulate CD80 or CD86 expression levels on these cells. Phosphorothioated CpG oligodeoxynucleotide 1668 was from Geneworks (Hindmarsh, Australia). Ovalbumin (OVA) and *E. coli* Lipopolysaccharide (LPS) were from Sigma (St. Louis, MO, USA). The SIINFEKL was synthesized by the Proteomics facility of the *Instituto de Salud Carlos III* using a peptide synthesizer (model 433A; Applied Biosystems, Foster City, CA, USA) and purified by reverse-phase HPLC. The SIIGFEKL and the Catnβ1 (β-catenin 329–336, RTYTYEKL) were purchased from Peptide2.0 Inc. (Chantilly, VA, USA). All cell line cultures and *in vitro* cultures were performed in complete medium (RPMI medium 1640 supplemented with 10% FCS, 50 μM 2-mercaptoethanol, 2 mM L-glutamine, 100 units/mL penicillin, and 100 μg/mL streptomycin) at 37°C.

**Figure 5:**
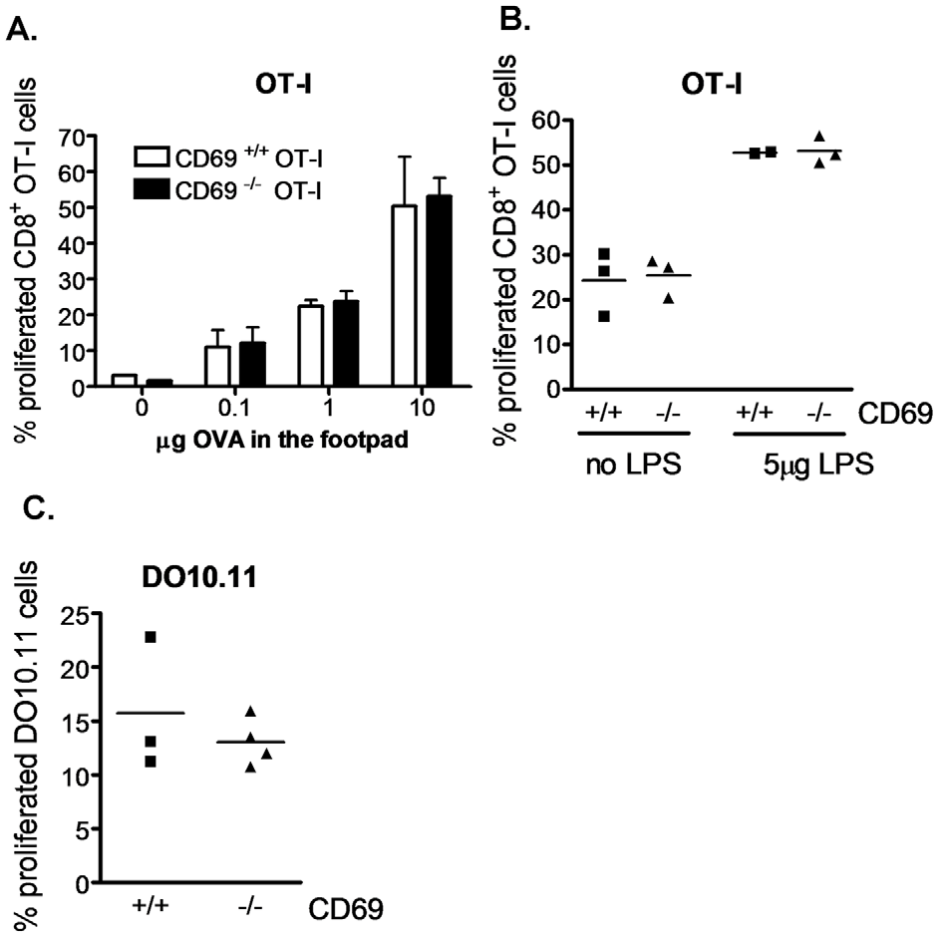
CD69 deficiency on T cells does not affect Ag-specific T cell proliferation in Ag-draining LN. **A.** Purified CD69^+/+^ or CD69^−/−^ OT-I CD8^+^ T cells were CFSE stained and transferred into recipients receiving the indicated doses of OVA and 5 µg of LPS subcutaneously in a posterior footpad. The percentage of proliferated OT-I CD8^+^ T cells was analyzed 42h later in the popliteal LN. Pool of two experiments, with 1 (dose 0) to 4 (doses 0.1–10 µg) mice per point. Bars represent SD. **B.** As in A, but mice received 10 µg of OVA with or without 5 µg of LPS in the footpad. **C.** Purified CD69^+/+^ or CD69^−/−^ RAG2^−/−^ DO10.11 CD4^+^ T cells were transferred into Balb/c mice receiving 1 µg of OVA subcutaneously in the footpad. 3 days later the popliteal LN were analyzed for the percentage of proliferated cells within DO10.11 CD4^+^ T cells.

### Flow cytometry

LN and spleen cells were treated with anti-CD16/32 (Fc-block 2.4G2, BD Biosciences, Franklin Lakes, NJ), and 7-Aminoactinomycin D (7AAD, BD Pharmingen) or Propidium Iodide (Molecular Probes, Eugene, OR, USA) were added in order to exclude dead cells. The following antibodies against mouse surface antigens were used: anti-CD4 (GK1.5), -CD8 (YTS 169.4), -CD11c (N418), -CD40 (FGK45.5), -B220/CD45RA (14.8), -CD69 (H1.2F3), -CD86 (PO3.1), and -Bst2 (120G8), all in-house produced; -CD4 (GK1.5), -CD8 (53–6.7), -CD25 (PC61.5), -Vα2 TCR (B20.1), and -DO10.11 TCR (KJ1), from eBioscience (San Diego, CA, USA). Cells were analyzed on FACScan, FACScalibur, FACScanto or LSRII flow cytometers (Becton Dickinson, Franklin Lakes, NJ USA), using CELLQuest or BD FacsDiva software (Becton Dickinson) and data was analyzed with FlowJo (Tree Star Inc., Ashland, OR, USA). In the experiments where the Mean Fluorescence Intensity (MFI) is represented, this parameter corresponds to the Geometric Mean.

**Figure 6:**
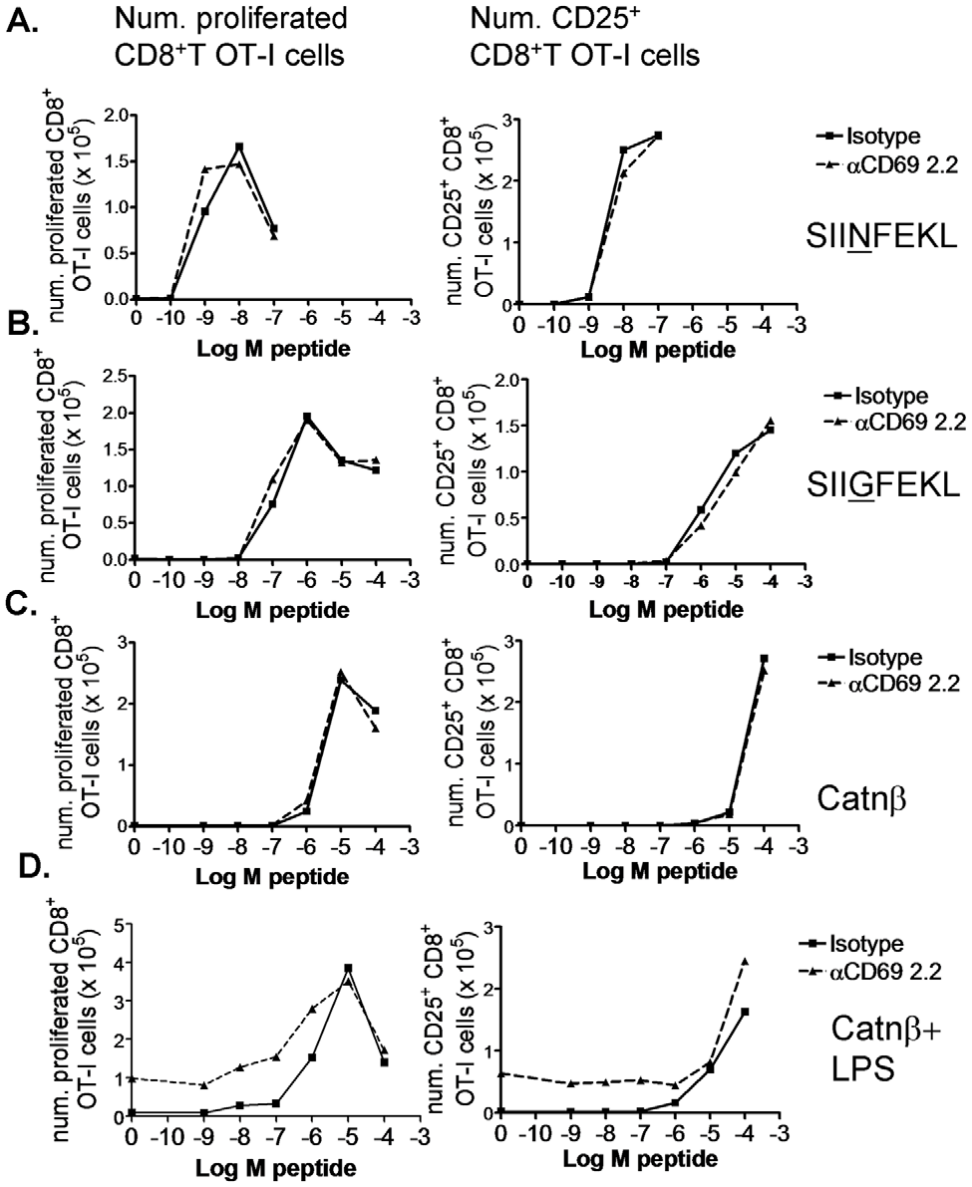
CD69 targeting does not alter CD8^+^ T cell priming threshold at different peptide agonistic affinities. Whole LN and spleen OT-I RAG1^−/−^ cells were stained with 2 µM CFSE and cultured at 10^6^ cells per well with the indicated doses of SIINFEKL (A), SIIGFEKL (B), Catnβ1 (C) and Catnβ1 plus 1 µg/ml of LPS (D), and 10 µg/ml of anti-CD69 2.2 mAb (dashed line) or isotype control (solid line). Graphs showing the number of proliferated (left column) and of CD25^+^ (right column) CD8^+^ T cells per well. Results representative of two experiments.

**Figure 7:**
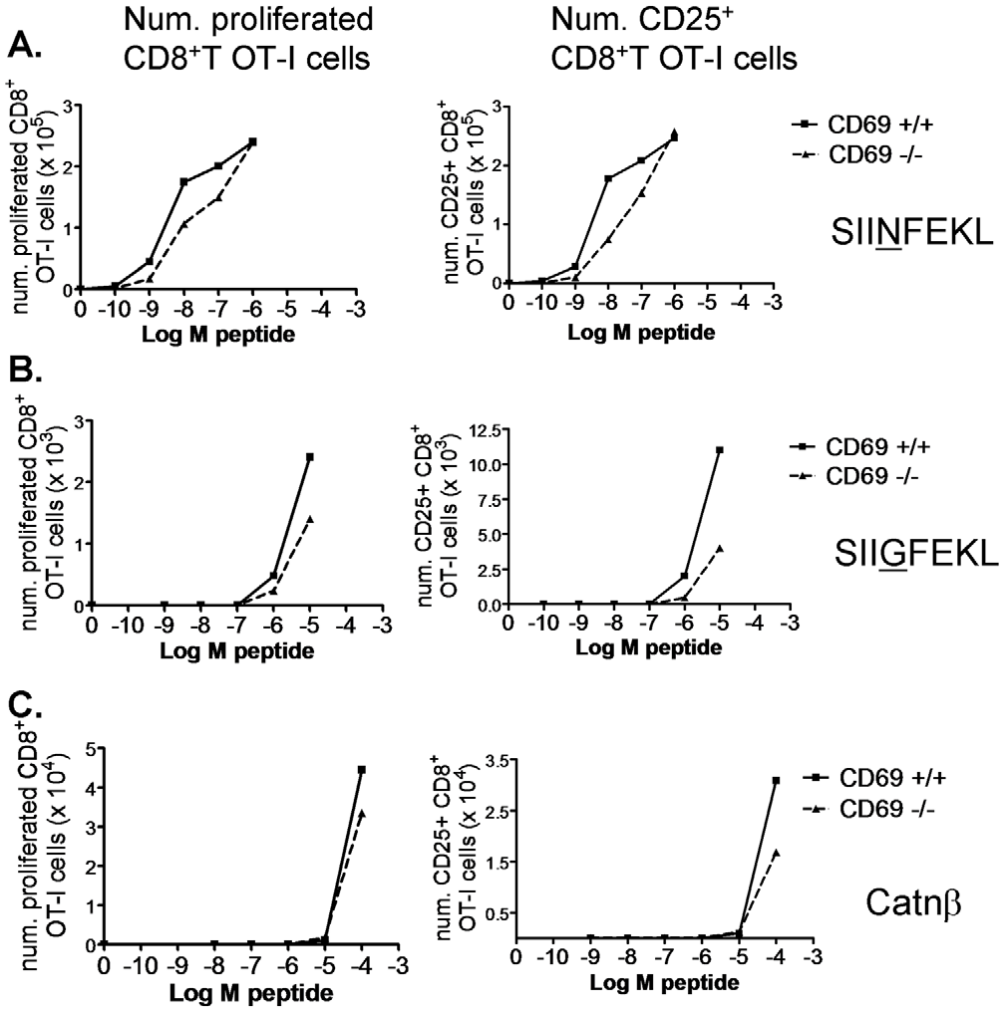
CD69 deficiency does not alter CD8^+^ T cell priming threshold at different peptide agonistic affinities. Spleen CD8^+^ T cells were purified from CD69^+/+^ (solid line) or CD69^−/−^ (dashed line) OT-I mice, stained with 2 µM CFSE and cultured at 0.5×10^6^ at 10∶1 with APC in the presence of the indicated doses of SIINFEKL (A), SIIGFEKL (B), and Catnβ1 (C) peptides. Graphs showing the number of proliferated (left column) and of CD25^+^ (right column) CD8^+^ T cells per well. The results are representative of two similar experiments with similar results.

### BMDC generation

Bone marrows were lysed and cultured in complete medium. For GM-CSF-derived BMDC, cells were cultured at 0.5×10^6^/ml in 24 well plates in the presence of 20 ng/mL Granulocyte and Macrophage Colony Stimulating factor (GM-CSF) (Immunotools, Friesoythe, Germany) for 6 days, changing half of the media for fresh media every two days. For Fms-related tyrosine kinase 3 ligand (Flt3l)-derived BMDC, cells were cultured at 1.5×10^6^/ml in 24well plates supplemented with in-house produced Flt3l for 9 days.

**Figure 8:**
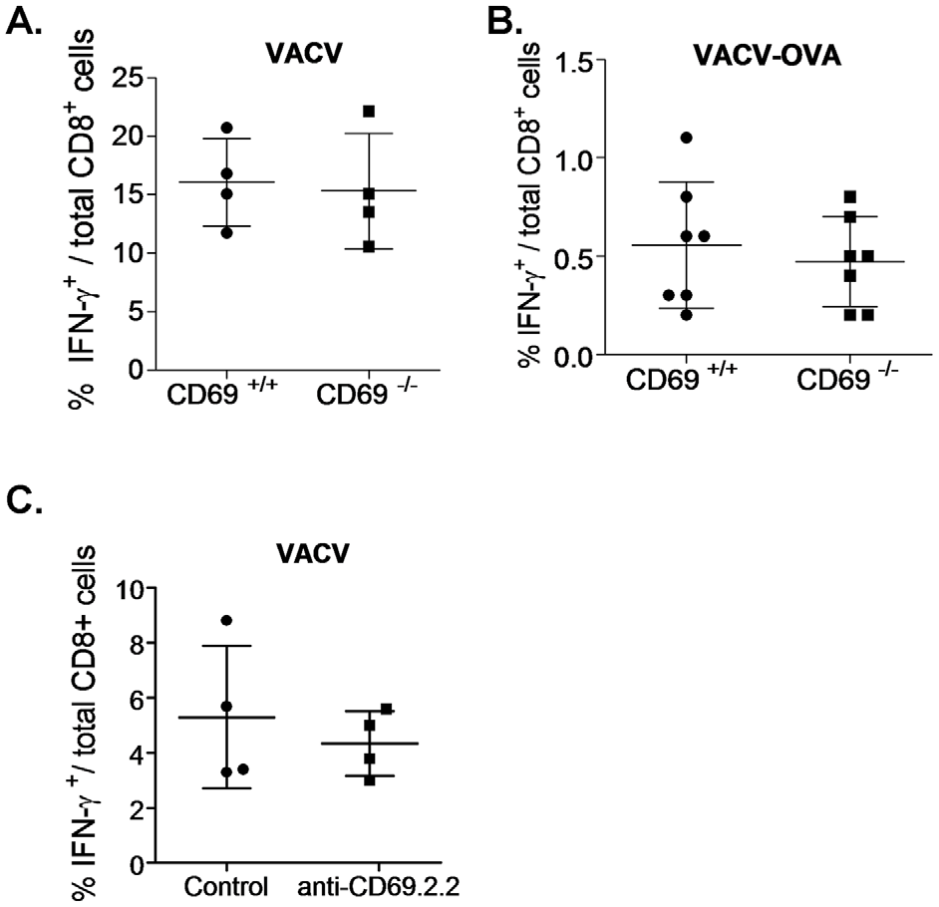
CD69 does not affect the primary formation of Vaccinia virus-specific CD8^+^ T cell populations. **A.** CD69^+/+^ or CD69^−/−^ mice were infected with VACV-WR and 7 days later spleen cells were reestimulated with uninfected (background control) or VACV-WR-infected RMA cells. **B.** CD69^+/+^ or CD69^−/−^ mice were infected with VACV-OVA and 7 days later spleen cells were reestimulated with or without (background control) SIINFEKL peptide. Pool of two experiments. **C.** H-2 class I knockout HLA-A*0201-transgenic mice were i.v. treated with 100 µg of anti-CD69 2.2 or left untreated, and were subsequently infected with VACV-WR. After 7 days, spleen cells were reestimulated with uninfected (background control) or VACV-WR-infected HLA-A*0201 transfectant RMA cells. In all cases, cells were stained for intracellular IFNγ, and the percentage of IFNγ^+^ CD8^+^ T cells within total cells was assessed. The background control values were substracted from each reestimulated sample value.

### DC Isolation

Splenic DC were isolated as described [36]. Briefly, C57BL/6 spleens were digested with DNase I (0.1%, Roche Molecular Biochemicals, Mannheim, Germany) and Collagenase (type II, Worthington Biochemical, Lakewood, NJ, USA), and centrifuged over Nycodenz medium (density 1.082 g/cm^3^, 1700× *g* for 10 min). The light-density fraction was treated with anti-CD3 (KT3-1.1), anti-Thy1-1 (T24/31.7), anti-Ly6G (IA8), anti-CD19 (ID3) and anti-erythrocyte (TER119), all home-produced, followed by immunomagnetic depletion with sheep anti-ratIgG coated Dynabeads (Dynal Biotech, Oslo, Norway). Where indicated, DC were stained with home produced anti-CD11c, -CD45RA and -Bst2 and further purified into conventional DC (cDC, CD11c^hi^ CD45RA^−^ Bst2^−^) or plasmacytoid DC (pDC, CD11c^int^ CD45RA^+^ Bst2^+^) with a FACS Aria sorter (Becton Dickinson).

### *In vitro* cultures

For T-DC co-cultures, OT-I and OT-II LN T cells were negatively selected using MAbs against CD11b (M1/70), F4/80, Ter-119, Gr-1 (RB6), MHC class II (M5/114), and CD4 (GK 1.5) or CD8 (YTS 169.4), respectively, followed by incubation with anti-rat IgG-coupled magnetic beads (Dynal Biotech). T cell preparations were 90–95% pure. T cells were stained with 5 µM carboxyfluorescein succinimidyl ester (CFSE). DC were plated at 5×10^3^ per well in U-bottom 96 well plates and cultured in complete media with the indicated conditions, in duplicate. Where indicated anti-CD69 2.2 or IgG1 control were added at 10 µg/ml. DC were co-cultured with 5×10^4^ OT-I CD8^+^ or OT-II CD4^+^ per well for 2 or 3 days respectively. After that, cells were stained with anti-Vα2, PI and anti-CD8 or anti-CD4 MAbs. For cultures of OT-I RAG1^−/−^ cells with peptide doses, whole LN and spleen cells were labeled with 2 µM CFSE and plated at 10^6^ cells per well in 96 well flat-bottom plates and were cultured with the indicated peptide doses, 10 µg/ml of anti-CD69 2.2 or IgG1 and with or without 1 µg/ml LPS for 3 days. For cultures of CD69^+/+^ or CD69^−/−^ OT-I cells with peptide doses, CD8^+^ T cells were negatively purified using CD8^+^ T cell isolation Kit and MACs columns (Miltenyi Biotec, Auburn, CA, USA), stained with 2 µM CFSE and cultured at 0.5×10^6^ cells per well together with 0.5×10^5^ CD69^+/+^ antigen presenting cells (the positive fraction) in 96 well flat-bottom plates with the indicated peptide doses for 3 days. Cells were then stained for CD8 and CD25 and analyzed by flow cytometry. 2.5×10^4^ Callibrite beads (Becton Dickinson) were added to each sample, and samples were acquired until 5x10^3^ beads had been collected.

### *In vivo* cell transfers

OT-I CD8^+^ T cells or DO10.11 CD4^+^ T cells were purified from CD69^+/+^ or CD69^−/−^ OT-I or DO10.11 mice, respectively, with CD8^+^ or CD4^+^ T cell isolation Kit and MACs columns (Miltenyi Biotec). After that, they were stained with 5 µM CFSE and injected intravenously into C57BL/6 or Balb/c recipients, respectively. Transferred recipients were anesthetized and received a subcutaneous injection in a posterior hind-footpad of the indicated OVA doses in a 10 µl volume using a Hamilton syringe (Hamilton Bonaduz, Bonaduz, Switzerland). At the indicated times, popliteal LN were collected, stained with anti-CD8^+^ or anti-CD4^+^ MAbs, and transferred cells were analyzed for CFSE dilution.

### Vaccinia virus infection and *ex vivo* intracytoplasmic staining (ICS)

Infections and ICS were performed as described [37]. Briefly, mice were infected with VACV-WR [38] or VACV-OVA_257–264_ (encoding the miniprotein MSIINFEKL) [39] with an intraperitoneal injection of 10^7^ plate forming units. After 7 days, spleens were harvested. For VACV-WR-infected mice, 10^6^ spleen cells were reestimulated with either RMA or RMA-S HLA-A*0201 cells infected with VACV-WR (at a multiplicity of infection of 10 plate forming units per cell for 2 h at 37°C, and washed), in the presence of brefeldin A (5 µg/ml). For VACV- OVA_257–264_-infected mice, 3×10^6^ spleen cells were reestimulated for 4h with SIINFEKL in the presence of brefeldin A (5 µg/ml). Cells were then surface-stained with anti-CD8 MAb (ProImmune, Oxford, United Kingdom), fixed with 4% paraformaldehide, washed, and incubated with anti-IFNγ MAb (BD PharMingen, San Diego, CA) in the presence of 0.1% saponin for 20 min at 4°C. Events were acquired and analyzed by flow cytometry as described.

## Results

### CD69 is upregulated on DC upon activation

CD69 upregulation on T cells upon specific Ag encounter has been well documented. Instead, CD69 expression by the other prototypic cell type implicated in T cell priming, the DC, has not been extensively studied. We have recently described certain constitutive CD69 expression by lymph node (LN) and spleen plasmacytoid DC (pDC) and by LN conventional DC (cDC) at steady state [34]. Here, we assess activation-induced CD69 upregulation on different DC subsets, activating them with Toll-like Receptors (TLR) ligands. CD69 was upregulated on both splenic pDC and cDC upon CpG olidodeoxynucleotide stimulation (Fig. 1A) in a dose-dependent manner (Fig. 1C), and it was prominent as soon as 3 hours after activation (Fig. 1B). CD69 upregulation was much higher in pDC than in the cDC subsets, which has been observed previously [40], and within the cDC subset, it was more prominent in CD8^+^ cDC than in CD8^−^ cDC (Fig. 1C). After 12 hours culture in the absence of CpG, there was also a marked CD69 upregulation on splenic DC (Fig. 1C), consistent with the activation that *ex vivo* purified DC undergo in culture [41]. CD69 was not expressed by unstimulated Granulocyte-Macrophage Colony Stimulating Factor (GM-CSF) and Fms-related tyrosine kinase 3 ligand (Flt3l)-derived Bone Marrow DC (BMDC), but it was upregulated on GM-CSF BMDC when we treated them with Lipopolysaccharide (LPS) (Fig. 2A) and on Flt3l BMDC when we treated them with CpG (Fig. 2D), in both cases in a dose dependent manner (Fig. 2B, E). Therefore, CD69 is upregulated on endogenous DC upon activation with TLR ligands, and this induced expression can be reproduced in *in vitro*-generated BMDC models.

### CD69 deficiency or targeting on DC do not affect their costimulation or Ag-presentation capacity

We next aimed at studying whether this CD69 expression by DC can have a role in T cell priming by putatively influencing costimulation or Ag presentation. To this end, we analyzed the effect of DC CD69 deficiency on the extent of the proliferation undergone by Ag-specific T cells, as well as on the expression of costimulatory molecules by DC. CD69 deficiency did not affect CD86 or CD40 expression on CpG-activated Flt3l-derived BMDC (Fig. 3A), and did not alter Ag-specific T cell proliferation in *in vitro* co-cultures of ovalbumin (OVA)-pulsed CD69^+/+^ or CD69^−/−^ Flt3l-derived BMDC with OVA-specific, transgenic OT-I CD8^+^ or OT-II CD4^+^ T cells (in the presence of CpG to induce CD69 expression) (Fig. 3B, C). We also analyzed whether MAb-based engagement of CD69 expressed on DC could have an effect on their Ag-presenting capacity. CD69 targeting with the anti-CD69 2.2 MAb in *in vitro* co-cultures of purified DC and OT-II cells in the presence of CpG and various OVA doses did not affect the OT-II proliferative response (Fig. 4B). In the co-culture of anti-CD69 2.2-treated pDC with OT-II cells (Fig. 4C) the proliferation was also unaltered. The treatment of cDC (containing the cross-presenting CD8^+^ DC subset) with anti-CD69 2.2 in the presence of CpG did not alter their cross-priming of OT-I cells (Fig. 4D). Consistently, treatment of CpG pre-activated Flt3l-derived BMDC with anti-CD69 2.2 did not affect the surface expression of CD86 and CD40 (Fig. 4A). Thus, CD69 expression or CD69 targeting on DC does not affect their costimulation or Ag presentation capacity to Ag-specific T cells.

### CD69 deficiency on T cells does not affect Ag-specific T cell proliferation in Ag-draining LN *in vivo*

CD69 has been well documented to be upregulated on T cell in response to the encounter with specific Ag. We thus wanted to assess whether CD69 could function as a costimulatory molecule on T cells. To analyze the effect of T cell CD69 deficiency on Ag priming *in vivo*, we transferred CD69^+/+^ or CD69^−/−^ OT-I cells into recipients receiving OVA subcutaneously into the footpad. In this setting, CD69 deficiency on T cells did not affect T cell proliferation in the popliteal LN of recipients injected with various OVA doses plus LPS (Fig. 5A) or 10 µg of OVA with or without LPS (Fig. 5B). A similar observation was made using the DO10.11 transfer model, in which CD69 deficiency did not affect proliferation of transferred OVA-specific transgenic DO10.11 CD4^+^ T cells in popliteal LN of recipients receiving OVA subcutaneously in the footpad (Fig. 5C). These results suggest that CD69 deficiency on T cells does not affect the extent of their Ag-specific priming *in vivo*.

### CD69 MAb-targeting or gene deficiency on Ag-specific CD8^+^ T cells do not alter the activation threshold at varying peptide agonistic levels *in vitro*

We further investigated the effect of CD69 targeting and deficiency on the extent of proliferation in *in vitro* cultures of transgenic CD8^+^ T cells with different agonist peptides.

The anti-CD69 2.2 MAb has been shown to bind to TCR-activated T cells [28]. However, the addition of this antibody to whole LN and spleen OT-I RAG1^−/−^ cultures with growing SIINFEKL peptide doses did not affect the dose from which T cell proliferation and CD25 upregulation started (Fig. 6A). This is consistent with our recently published *in vivo* results, in which anti-CD69 2.2 treatment does not alter proliferation of OT-I transferred T cells in Ag-draining LN of OVA-bearing mice [34]. Reasoning that SIINFEKL might be a too strong agonist and that a role in costimulation would be better noticed using weaker agonist peptides, we used the SIIGFEKL peptide variant and the Catnβ1 peptide (an endogenous peptide from the β-catenin protein mediating positive thymic selection of the OT-I clone). In both cases, anti-CD69 2.2 MAb addition neither affected the activation threshold, the extent of the proliferative response, nor CD25 expression (Fig. 6B, C). We have also recently shown that anti-CD69 2.2 induces bystander T cell proliferation *in vivo* and *in vitro*, dependent on IL-2 production by pDC and CD25 upregulation by T cells. *In vitro*, for CD8^+^ T cells, this bystander proliferation was observed when LPS was added to whole LN and spleen cultures. It is unclear whether bystander proliferation depends on the contact of the TCR with MHC molecules presenting endogenous weak agonist peptides. In an attempt to address this question, we added LPS to OT-I RAG1^−/−^ cultures with Catnβ1 peptide. If the anti-CD69 2.2-induced bystander proliferation was dependent on the recognition of endogenous weak agonist peptides, the difference in proliferation between the isotype control and the anti-CD69 2.2-treated samples would be higher at higher Catnβ1 peptide occupancy of the MHC-I, that is, at higher Catnβ1 peptide doses. However, this difference was already maximal in the absence of peptide, was maintained through a range of growing peptide doses, and disappeared at peptide doses giving maximal proliferation (Fig. 6D). This suggests that this bystander proliferation might be rather independent of TCR recognition of endogenous weak agonist ligands. In agreement with this hypothesis, anti-CD69 2.2 treatment induced bystander proliferation of DO10.11 CD4^+^ T cells when these were transferred into CIITA^−/−^ recipients, which have deficient MHC-II expression, to the same extent as when they were transferred into WT mice (our unpublished data). In Fig. 6D, as expected from previous results, in the presence of LPS and in the absence of peptide or at low peptide doses, the anti-CD69 2.2 induced CD25 upregulation on CD8^+^ T cells. Similarly to CD69 targeting, we did not find a significant effect of CD69 deficiency on the CD8^+^ T cell activation threshold, as measured by the peptide dose from which T cell proliferation and CD25 upregulation start to be apparent (even though the extent of proliferation and CD25 upregulation were slightly lower for CD69^−/−^ OT-I cells than for CD69^+/+^ OT-I cells). This was true for the three peptides tested (Fig. 7). These results point to that CD69 does not function as a costimulatory molecule on T cells.

### Neither CD69 targeting nor CD69 deficiency affect the formation of virus-specific CD8^+^ T cell populations in the primary immune response to Vaccinia virus infection

To check for a possible role of CD69 expressed by either DC or T cells on T cell priming in a physiological model of infection, in which more factors and possible indirect effects can also play a role, we used various experimental settings of the Vaccinia virus (VACV) infection model. CD69 deficiency of VACV-WR (wild type) (Fig. 8A) or VACV-OVA (Fig. 8B) -infected mice did not alter the formation of the VACV- or SIINFEKL-specific CD8^+^ T cell pools, respectively, as measured by the percentage of IFNγ producing spleen CD8^+^ T cells in response to VACV or SIINFEKL reestimulation at the peak of the primary immune responses. To test for the effect of anti-CD69 2.2 treatment on the anti-Vaccinia virus CD8^+^ T cell response, we took advantage of the availability of the H-2 class I knockout HLA-A*0201-transgenic mouse model, whose surface MHC-I expression is only the one of HLA-A2. In these mice, the MAb treatment did not affect the percentage of CD8^+^ T cells responding to HLA-A2-presented Vaccinia virus antigens either (Fig. 8C). These results indicate that neither CD69 targeting nor CD69 deficiency affects the primary expansion and formation of Ag-specific CD8^+^ T cell pools, at least in the Vaccinia virus infection model.

## Discussion

CD69 has been found to be rapidly upregulated on all the leukocyte lineages studied, upon activation with the corresponding stimuli. When testing whether this was also the case for DC, we found that CD69 is upregulated on this cell type as soon as 3 hours after addition of TLR ligands. Thus, the expression pattern of CD69 on both T cells and DC is reminiscent of the one of costimulatory molecules. Some C-type lectins are upregulated upon activation on T cells, and have a costimulatory or coinhibitory role on Ag-driven T cell activation, influencing, among other parameters, the proliferative response. Also, C-type lectins expressed on DC can initiate signaling or modulate TLR signaling, affecting DC maturation and thus, possibly altering their Ag presentation and costimulation abilities. In this work we have examined a possible role for CD69 in the mentioned processes through analyzing the effect of CD69 targeting and deficiency on the extent of T cell priming.

*In vitro*, we did not observe any effect of CD69 deficiency or targeting of DC using different DC types, OT-I or OT-II responders and graded OVA doses. In addition, neither CD69 targeting nor its deficiency on T cells influenced the minimal peptide dose needed for a proliferative response of OT-I T cells to different peptides, not even to the weak agonist ones. In contrast to CD69 deficiency, the deficiency of costimulatory molecules such as CD28 has been observed to lead to the lack of T cell priming to weak agonist peptides, even at high doses [42]. Consistent with the *in vitro* results, in *in vivo* adoptive transfers of transgenic OT-I CD8^+^ or DO10.11 CD4^+^ T cells into recipient mice receiving OVA subcutaneously in the footpad, CD69 deficiency did not affect the extent of the Ag-driven T cell proliferation in the Ag draining LNs. By parallel experimental approaches, we have previously reported that CD69 deficiency [10] or anti-CD69 2.2 treatment [34] of the recipient mice do not affect T cell cross-priming. All these data point to that CD69 does not quantitatively affect the priming of Ag-specific T cells.

In a recent work we have shown that, in the absence of specific Ag, CD69 targeting with the anti-CD69 2.2 MAb induces bystander T cell proliferation dependent on IL-2 and on CD25 upregulation on T cells (*in vitro*, for CD8^+^ T cells, it needed the addition of LPS). This might seem contradictory with the results shown in the present work, and also with the results in that same work showing that anti-CD69 2.2 *in vivo* treatment did not affect Ag-specific T cell proliferation, since one might expect that the increased IL-2 and CD25 expression would lead to increased Ag-specific proliferation. However it is possible that in the presence of specific Ag, the TCR signaling itself already induces the production of such IL-2 and CD25 amounts that overrule the ones induced by CD69 targeting. Alternatively, in the presence of TCR ligation, CD69 could be uncoupled from the downstream signaling events leading to the bystander proliferative effect. These hypotheses are consistent with the data shown in Fig. 5D, in which the addition of LPS allows anti-CD69 2.2-induced bystander T cell proliferation in the absence of Ag. In this experiment, the difference of proliferation versus the wells with isotype control is already maximal in the absence of peptide, is maintained during a range of growing specific peptide doses, suggesting that the bystander proliferation is added on top of the Ag-specific proliferation, and disappears at the dose that gives a maximal response.

Our results are in contrast with the initial *in vitro* data showing that anti-CD3 or PMA-activated human T cells were further induced to proliferate by CD69 targeting, but are in agreement with a posterior observation indicating that Ag-specific T cell proliferation was unaffected in CD69^−/−^ mouse T cells *in vivo* [27]. Of notice, not all the anti-CD69 MAbs tested in initial works were reported to have this proliferation-enhancing effect on human T cells [43]–[44]. Altogether, more physiological *in vitro* and *in vivo* data argue against the initially proposed role for CD69 as a costimulatory molecule.

When studying the influence of CD69 in a physiological setting in which the priming occurs to endogenous naïve T cell pools of various frequencies and TCR affinities, such as the Vaccinia virus infection, we observed that CD69 targeting or deficiency did not alter the size of the VACV-specific T cell population at the peak of the primary response. This is in support of our previous hypothesis that, if we found a slightly smaller population of Lm-specific T cells in Lm-infected CD69^−/−^ mice [10], it was not due to an intrinsic defect in Ag-specific T cell priming. Instead, it could be a collateral effect of the Lm-induced, type I IFN-mediated massive lymphocyte apoptosis [45] affecting also primed Lm-specific T cells. Of notice, the defect in the control of Lm infection in CD69^−/−^ mice was noticeable as soon as day 1 post-infection (and thus, induced by differences in the innate immune response), was associated to increased type I IFN levels and increased spleen cell death, and was not observed in lymphocyte deficient mice. In contrast to Lm infection, Vaccinia virus infection does not lead to massive lymphocyte apoptosis, and this might be the reason for it not inducing this innate immunity-based difference in the size of specific T cell populations.

Altogether, the *in vivo* and *in vitro* data point to that targeting of CD69 expressed or up-regulated on DC does not affect their Ag processing, Ag presentation or costimulation capacity, and that CD69 does not function as a costimulatory molecule on DC or on T cells. These results do not rule out, though, that CD69 could affect other important aspects influencing or being determined by DC-T cell interaction, such as DC polarization or T cell polarization and programming. In this regard, CD69 has recently been reported to inhibit Th17 differentiation in Ag primed CD4^+^ T cells [46]. Taking this into account, it could be hypothesized that the exacerbated forms of Ag-specific T cell-dependent diseases reported in CD69^−/−^ and anti-CD69 2.2-treated mice are not due to differential priming of Ag-specific T cells but might rather be owed to skewed polarization of primed T cells.

On the whole, this work contributes to resolving a previous controversy and to shift the focus on CD69 towards its effect on T cell polarization, as it is starting to be apparent, rather than on the extent of T cell priming and T cell costimulation.
